# Supplementary material for: COZOID: contact zone identifier for visual analysis of protein-protein interactions
Source: BMC Bioinformatics. 2018 Apr 6;19:125. doi: 10.1186/s12859-018-2113-6 (PMC5889581; doi:10.1186/s12859-018-2113-6)
Supplement: Supplementary file 2 — Software build. Executable binary file of the software tool. (ZIP 118,784 kb) [file 12859_2018_2113_MOESM2_ESM.zip › cozoid/java/docs/beansbinding-1.2.1-doc.zip!/org/jdesktop/el/impl/parser/ELParser.html]

ELParser (Beans Binding)


---


|  |  |  |
| --- | --- | --- |
| **Overview** \| **Package** \| **Class** \| **Use** \| **Tree** \| **Deprecated** \| **Index** \| **Help** | |  |
| **PREV CLASS**   **NEXT CLASS** | **FRAMES**    **NO FRAMES**     **All Classes** |  |
| SUMMARY: NESTED \| FIELD \| CONSTR \| METHOD | DETAIL: FIELD \| CONSTR \| METHOD |  |


---


## org.jdesktop.el.impl.parser Class ELParser

```
java.lang.Object
  org.jdesktop.el.impl.parser.ELParser
```

**All Implemented Interfaces:**: ELParserConstants, ELParserTreeConstants

---

``` public class ELParser extends java.lang.Object implements ELParserTreeConstants, ELParserConstants ```

---

| **Field Summary** | |
| --- | --- |
| `Token` | `jj_nt` |
| `protected  org.jdesktop.el.impl.parser.JJTELParserState` | `jjtree` |
| `boolean` | `lookingAhead` |
| `Token` | `token` |
| `ELParserTokenManager` | `token_source` |

**Fields inherited from interface org.jdesktop.el.impl.parser.ELParserTreeConstants**

`JJTAND, JJTBRACKETSUFFIX, JJTCHOICE, JJTCOMPOSITEEXPRESSION, JJTDEFERREDEXPRESSION, JJTDIV, JJTDOTSUFFIX, JJTDYNAMICEXPRESSION, JJTEMPTY, JJTEQUAL, JJTFALSE, JJTFLOATINGPOINT, JJTFUNCTION, JJTGREATERTHAN, JJTGREATERTHANEQUAL, JJTIDENTIFIER, JJTINTEGER, JJTLESSTHAN, JJTLESSTHANEQUAL, JJTLITERALEXPRESSION, JJTMINUS, JJTMOD, JJTMULT, JJTNEGATIVE, jjtNodeName, JJTNOT, JJTNOTEQUAL, JJTNULL, JJTOR, JJTPLUS, JJTSTRING, JJTTRUE, JJTVALUE, JJTVOID`

**Fields inherited from interface org.jdesktop.el.impl.parser.ELParserConstants**

`AND0, AND1, BADLY_ESCAPED_STRING_LITERAL, COLON, COMMA, DEFAULT, DIGIT, DIV0, DIV1, DOT, EMPTY, END_EXPRESSION, EOF, EQ0, EQ1, EXPONENT, FALSE, FLOATING_POINT_LITERAL, FUNCTIONSUFFIX, GE0, GE1, GT0, GT1, IDENTIFIER, ILLEGAL_CHARACTER, IMPL_OBJ_START, IN_EXPRESSION, INSTANCEOF, INTEGER_LITERAL, LBRACK, LE0, LE1, LETTER, LITERAL_EXPRESSION, LPAREN, LT0, LT1, MINUS, MOD0, MOD1, MULT, NE0, NE1, NOT0, NOT1, NULL, OR0, OR1, PLUS, QUESTIONMARK, RBRACK, RPAREN, START_DEFERRED_EXPRESSION, START_DYNAMIC_EXPRESSION, STRING_LITERAL, tokenImage, TRUE`


**Constructor Summary**

`ELParser(ELParserTokenManager tm)`

`ELParser(java.io.InputStream stream)`

`ELParser(java.io.Reader stream)`


| **Method Summary** | |
| --- | --- |
| `void` | `And()` |
| `void` | `Boolean()` |
| `void` | `BracketSuffix()` |
| `void` | `Choice()` |
| `void` | `Compare()` |
| `AstCompositeExpression` | `CompositeExpression()` |
| `void` | `DeferredExpression()` |
| `void` | `disable_tracing()` |
| `void` | `DotSuffix()` |
| `void` | `DynamicExpression()` |
| `void` | `enable_tracing()` |
| `void` | `Equality()` |
| `void` | `Expression()` |
| `void` | `FloatingPoint()` |
| `void` | `Function()` |
| `ParseException` | `generateParseException()` |
| `Token` | `getNextToken()` |
| `Token` | `getToken(int index)` |
| `void` | `Identifier()` |
| `void` | `Integer()` |
| `void` | `Literal()` |
| `void` | `LiteralExpression()` |
| `void` | `Math()` |
| `void` | `Multiplication()` |
| `void` | `NonLiteral()` |
| `void` | `Null()` |
| `void` | `Or()` |
| `static Node` | `parse(java.lang.String ref)` |
| `void` | `ReInit(ELParserTokenManager tm)` |
| `void` | `ReInit(java.io.InputStream stream)` |
| `void` | `ReInit(java.io.Reader stream)` |
| `void` | `String()` |
| `void` | `Unary()` |
| `void` | `Value()` |
| `void` | `ValuePrefix()` |
| `void` | `ValueSuffix()` |

**Methods inherited from class java.lang.Object**

`clone, equals, finalize, getClass, hashCode, notify, notifyAll, toString, wait, wait, wait`

**Field Detail**

### jjtree

```
protected org.jdesktop.el.impl.parser.JJTELParserState jjtree
```

---


### token\_source

```
public ELParserTokenManager token_source
```

---


### token

```
public Token token
```

---


### jj\_nt

```
public Token jj_nt
```

---


### lookingAhead

```
public boolean lookingAhead
```


**Constructor Detail**

### ELParser

```
public ELParser(java.io.InputStream stream)
```

---


### ELParser

```
public ELParser(java.io.Reader stream)
```

---


### ELParser

```
public ELParser(ELParserTokenManager tm)
```


**Method Detail**

### parse

```
public static Node parse(java.lang.String ref)
                  throws ELException
```

:   **Throws:**: `ELException`

---


### CompositeExpression

```
public final AstCompositeExpression CompositeExpression()
                                                 throws ParseException
```

:   **Throws:**: `ParseException`

---


### LiteralExpression

```
public final void LiteralExpression()
                             throws ParseException
```

:   **Throws:**: `ParseException`

---


### DeferredExpression

```
public final void DeferredExpression()
                              throws ParseException
```

:   **Throws:**: `ParseException`

---


### DynamicExpression

```
public final void DynamicExpression()
                             throws ParseException
```

:   **Throws:**: `ParseException`

---


### Expression

```
public final void Expression()
                      throws ParseException
```

:   **Throws:**: `ParseException`

---


### Choice

```
public final void Choice()
                  throws ParseException
```

:   **Throws:**: `ParseException`

---


### Or

```
public final void Or()
              throws ParseException
```

:   **Throws:**: `ParseException`

---


### And

```
public final void And()
               throws ParseException
```

:   **Throws:**: `ParseException`

---


### Equality

```
public final void Equality()
                    throws ParseException
```

:   **Throws:**: `ParseException`

---


### Compare

```
public final void Compare()
                   throws ParseException
```

:   **Throws:**: `ParseException`

---


### Math

```
public final void Math()
                throws ParseException
```

:   **Throws:**: `ParseException`

---


### Multiplication

```
public final void Multiplication()
                          throws ParseException
```

:   **Throws:**: `ParseException`

---


### Unary

```
public final void Unary()
                 throws ParseException
```

:   **Throws:**: `ParseException`

---


### Value

```
public final void Value()
                 throws ParseException
```

:   **Throws:**: `ParseException`

---


### ValuePrefix

```
public final void ValuePrefix()
                       throws ParseException
```

:   **Throws:**: `ParseException`

---


### ValueSuffix

```
public final void ValueSuffix()
                       throws ParseException
```

:   **Throws:**: `ParseException`

---


### DotSuffix

```
public final void DotSuffix()
                     throws ParseException
```

:   **Throws:**: `ParseException`

---


### BracketSuffix

```
public final void BracketSuffix()
                         throws ParseException
```

:   **Throws:**: `ParseException`

---


### NonLiteral

```
public final void NonLiteral()
                      throws ParseException
```

:   **Throws:**: `ParseException`

---


### Identifier

```
public final void Identifier()
                      throws ParseException
```

:   **Throws:**: `ParseException`

---


### Function

```
public final void Function()
                    throws ParseException
```

:   **Throws:**: `ParseException`

---


### Literal

```
public final void Literal()
                   throws ParseException
```

:   **Throws:**: `ParseException`

---


### Boolean

```
public final void Boolean()
                   throws ParseException
```

:   **Throws:**: `ParseException`

---


### FloatingPoint

```
public final void FloatingPoint()
                         throws ParseException
```

:   **Throws:**: `ParseException`

---


### Integer

```
public final void Integer()
                   throws ParseException
```

:   **Throws:**: `ParseException`

---


### String

```
public final void String()
                  throws ParseException
```

:   **Throws:**: `ParseException`

---


### Null

```
public final void Null()
                throws ParseException
```

:   **Throws:**: `ParseException`

---


### ReInit

```
public void ReInit(java.io.InputStream stream)
```

---


### ReInit

```
public void ReInit(java.io.Reader stream)
```

---


### ReInit

```
public void ReInit(ELParserTokenManager tm)
```

---


### getNextToken

```
public final Token getNextToken()
```

---


### getToken

```
public final Token getToken(int index)
```

---


### generateParseException

```
public ParseException generateParseException()
```

---


### enable\_tracing

```
public final void enable_tracing()
```

---


### disable\_tracing

```
public final void disable_tracing()
```


---


|  |  |  |
| --- | --- | --- |
| **Overview** \| **Package** \| **Class** \| **Use** \| **Tree** \| **Deprecated** \| **Index** \| **Help** | |  |
| **PREV CLASS**   **NEXT CLASS** | **FRAMES**    **NO FRAMES**     **All Classes** |  |
| SUMMARY: NESTED \| FIELD \| CONSTR \| METHOD | DETAIL: FIELD \| CONSTR \| METHOD |  |


---
